# Early detection of a new pseudoaneurysm from an alternative feeder after emergent embolization for ruptured giant renal angiomyolipoma: A case report

**DOI:** 10.1016/j.eucr.2026.103597

**Published:** 2026-09-07

**Authors:** Takaaki Mori, Hajime Takaki, Yasunari Yamada, Yoshiyasu Sato, Tadamasa Shibuya, Yoshiki Asayama

**Affiliations:** a Department of Radiology, Oita Red Cross Hospital, Chiyo-machi, Oita, 870-0033, Japan; b Department of Urology, Oita Red Cross Hospital, Chiyo-machi, Oita, 870-0033, Japan; c Department of Radiology, Oita University Faculty of Medicine, Yufu, Oita, 879-5593, Japan

**Keywords:** Angiomyolipoma, Pseudoaneurysm, Transarterial embolization, Renal hemorrhage, Computed tomography

## Abstract

Renal angiomyolipomas (AMLs) may rupture and cause life-threatening hemorrhage. We report a man in his 40s with a ruptured giant renal AML who underwent emergent transarterial embolization after contrast-enhanced computed tomography demonstrated a pseudoaneurysm. Although initial hemostasis was achieved and he remained clinically stable, follow-up computed tomography performed 5 days later revealed a newly apparent 20-mm pseudoaneurysm arising from an alternative feeder, requiring repeat embolization. This case suggests that early postprocedural contrast-enhanced computed tomography may be useful in selected patients with ruptured giant or multifocal AMLs and high-risk vascular morphology.

## 1 Introduction

Angiomyolipomas (AMLs) are benign mesenchymal neoplasms that may nevertheless rupture and result in potentially life-threatening retroperitoneal hemorrhage.^1^ Although a tumor diameter of ≥4 cm has traditionally served as a pragmatic threshold for considering intervention, the risk of rupture is influenced not only by lesion size but also by the presence and dimensions of intratumoral aneurysms.^2^ Transarterial embolization (TAE) is an established kidney-preserving hemostatic intervention for ruptured AMLs and plays an important role in both acute hemorrhage control and long-term disease management.^3^ However, a subset of patients requires repeat intervention following initial TAE, underscoring the importance of appropriate imaging surveillance after hemostasis has been achieved.^4^ Herein, a case of ruptured giant renal AML is described in which emergent TAE achieved initial hemostasis and clinical stabilization; however, follow-up computed tomography (CT) performed 5 days later demonstrated a newly apparent pseudoaneurysm arising from a separate arterial feeder, necessitating repeat embolization. This case highlights that, in ruptured AMLs with high-risk morphologic characteristics, clinically occult vascular lesions supplied by alternative feeding arteries may become detectable over a short interval and raises the possibility that early postprocedural imaging surveillance may be useful in selected patients even in the setting of apparent clinical stability.

## 2 Case report

A man in his 40s with no known medical history, regular medications, or relevant comorbidities presented with a 2-day history of progressively worsening right flank pain. Computed tomography performed at the referring institution demonstrated an abdominal mass, prompting referral to our hospital for further evaluation. On presentation, vital signs were as follows: blood pressure, 116/77 mmHg; heart rate, 125 beats/min; respiratory rate, 22 breaths/min; oxygen saturation, 99% on room air; and temperature, 37.3°C. Physical examination revealed a soft abdomen without tenderness or rebound; however, right costovertebral angle tenderness was noted. Laboratory evaluation demonstrated a hemoglobin level of 9.5 g/dL, serum creatinine level of 1.33 mg/dL (estimated glomerular filtration rate, 47 mL/min/1.73 m^2^), platelet count of 153,000/μL, white blood cell count of 27,200/μL, and C-reactive protein level of 9.28 mg/dL. Urinalysis was positive for blood (±0.03). Peripheral intravenous access was established, and the patient received intravenous fluid resuscitation and transfusion of two units of packed red blood cells.

**Fig. 1:**
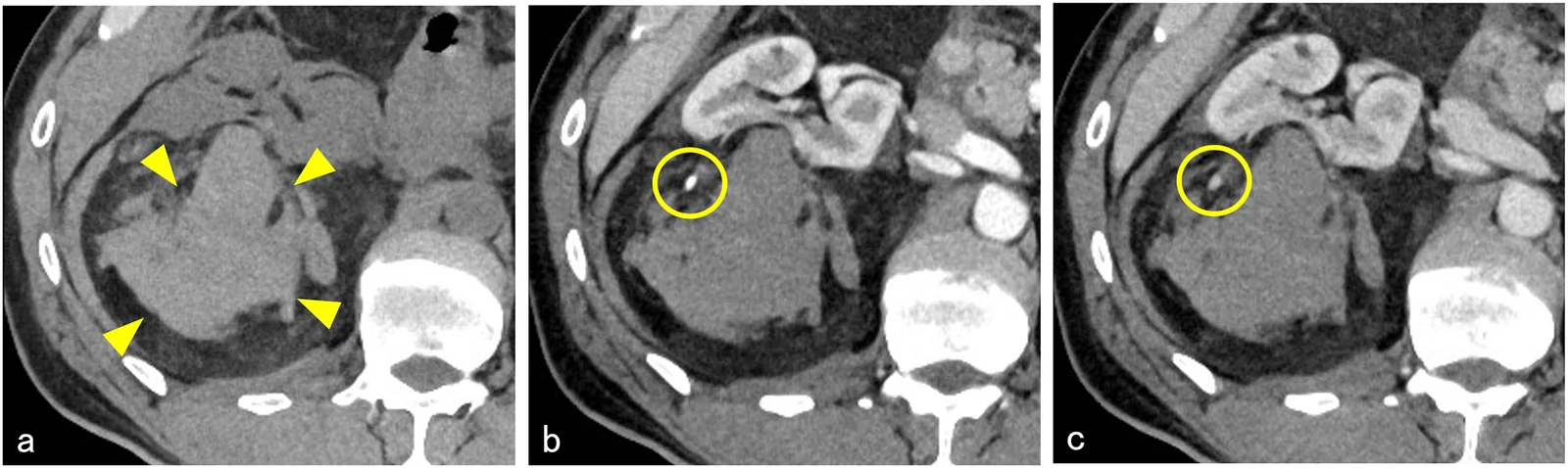
Initial CT findings. (a) Unenhanced CT image demonstrating hematomas associated with rupture of an angiomyolipoma (AML) in the dorsal aspect of the right kidney (arrowheads). (b) Arterial-phase contrast-enhanced CT image demonstrating an enhancing nodular lesion along the ventral aspect of the intratumoral hematoma, suspicious for a pseudoaneurysm and likely arising from the ventral branch of the right renal artery (yellow circle). (c) Equilibrium-phase contrast-enhanced CT image demonstrating persistent enhancement of the pseudoaneurysm along the ventral aspect of the intratumoral hematoma (yellow circle).

**Fig. 2:**
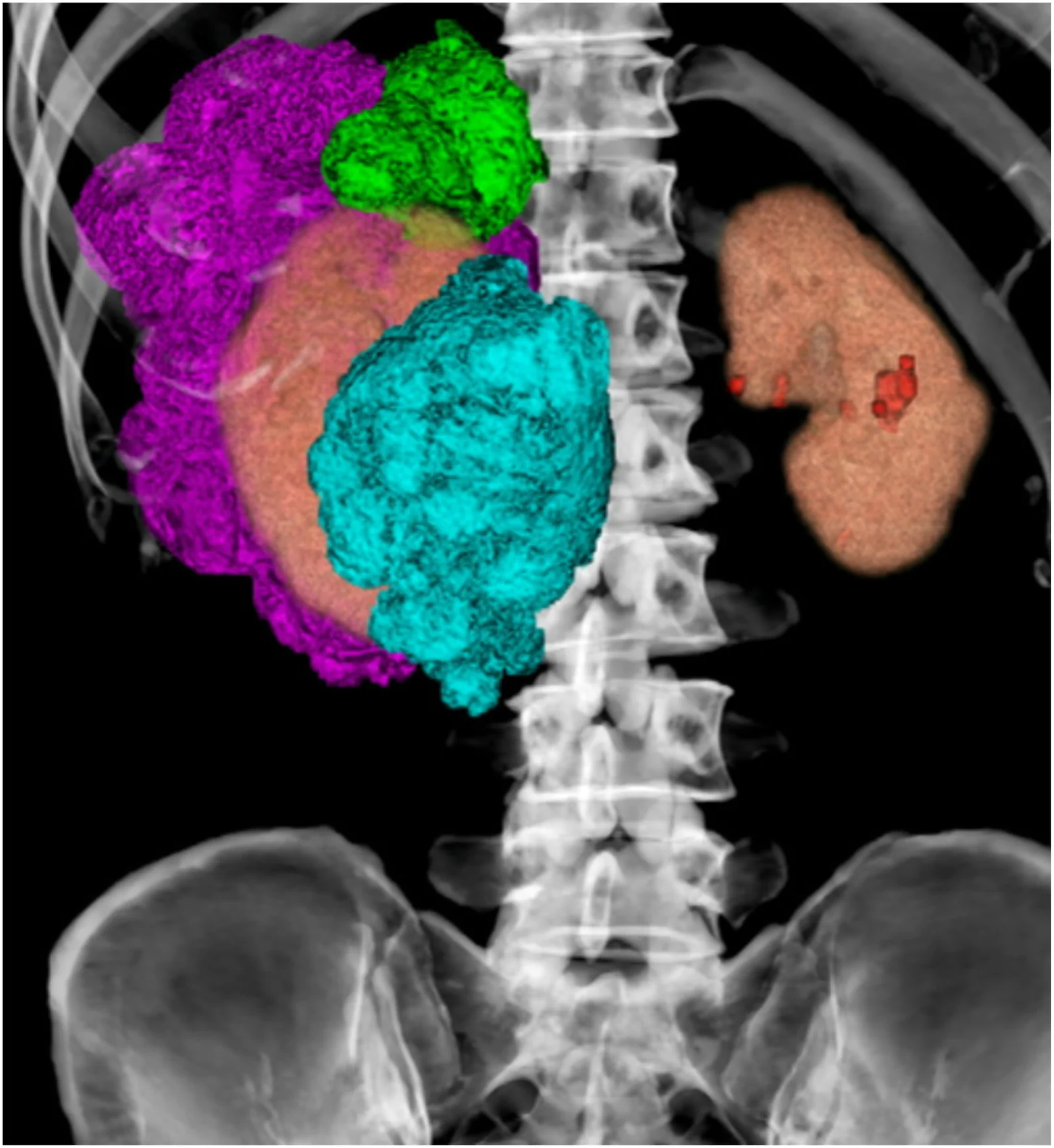
Volume-rendered CT image demonstrating bilateral multifocal renal angiomyolipomas. The three large right renal lesions are highlighted in pink, green, and light blue, whereas multiple small left renal lesions are shown in red.

Because non-contrast CT findings were suggestive of rupture of a right renal angiomyolipoma (AML), contrast-enhanced CT was performed immediately. Imaging demonstrated rupture of a dorsal AML in the right kidney measuring 10 cm in maximum diameter, accompanied by an intratumoral hematoma and a large surrounding peritumoral hematoma (Fig. 1a). Contrast-enhanced CT further demonstrated a 4-mm enhancing focus contiguous with a ventral renal arterial branch along the ventral margin of the intratumoral hematoma, suspicious for pseudoaneurysm formation. No definite active contrast extravasation was observed (Fig. 1b and c). Additional right renal AMLs measuring 8.5 cm and 7.6 cm were identified, and multiple millimeter-sized AMLs were scattered throughout the left kidney (Fig. 2).

**Fig. 3:**
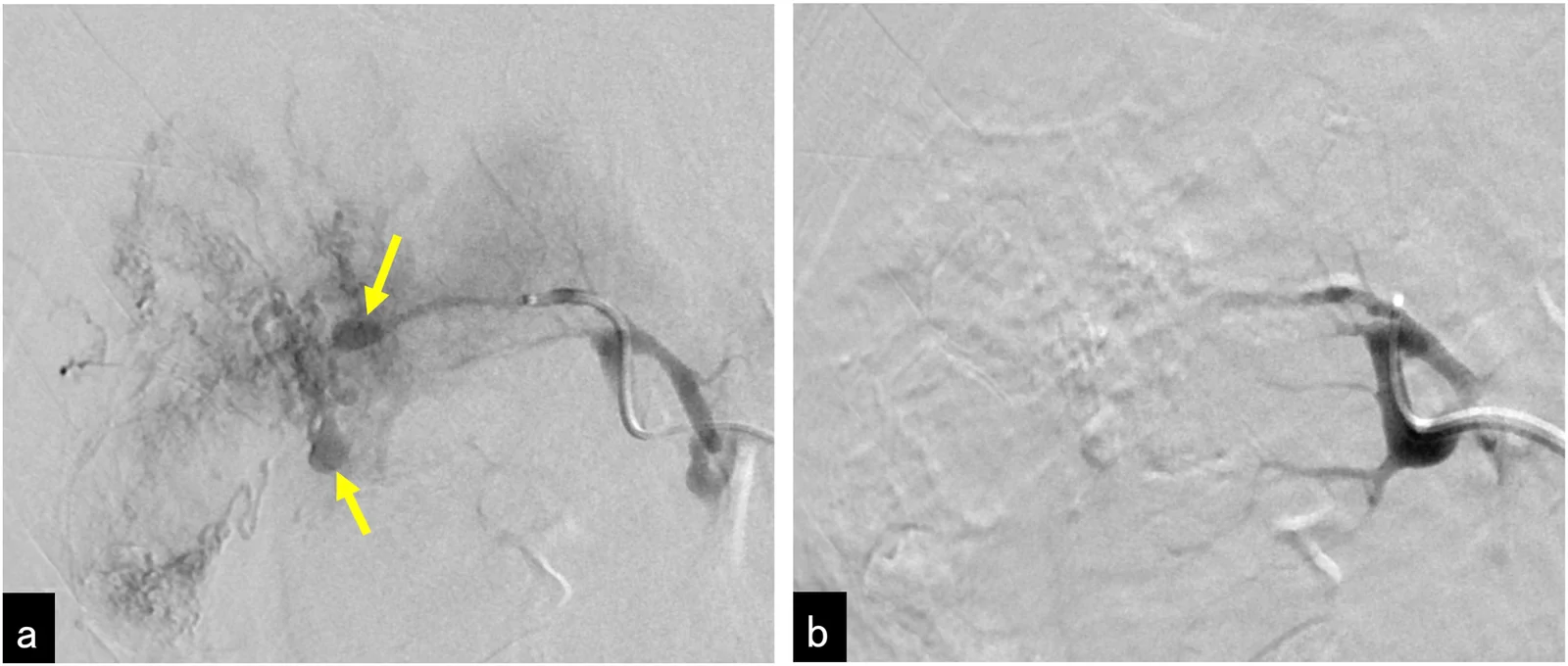
Initial angiography and embolization. (a) Selective angiography of the distal ventral branch of the right renal artery, considered the bleeding source, demonstrating pseudoaneurysms (arrows). (b) Repeat angiography after gelatin sponge embolization demonstrating disappearance of the pseudoaneurysms.

**Fig. 4:**
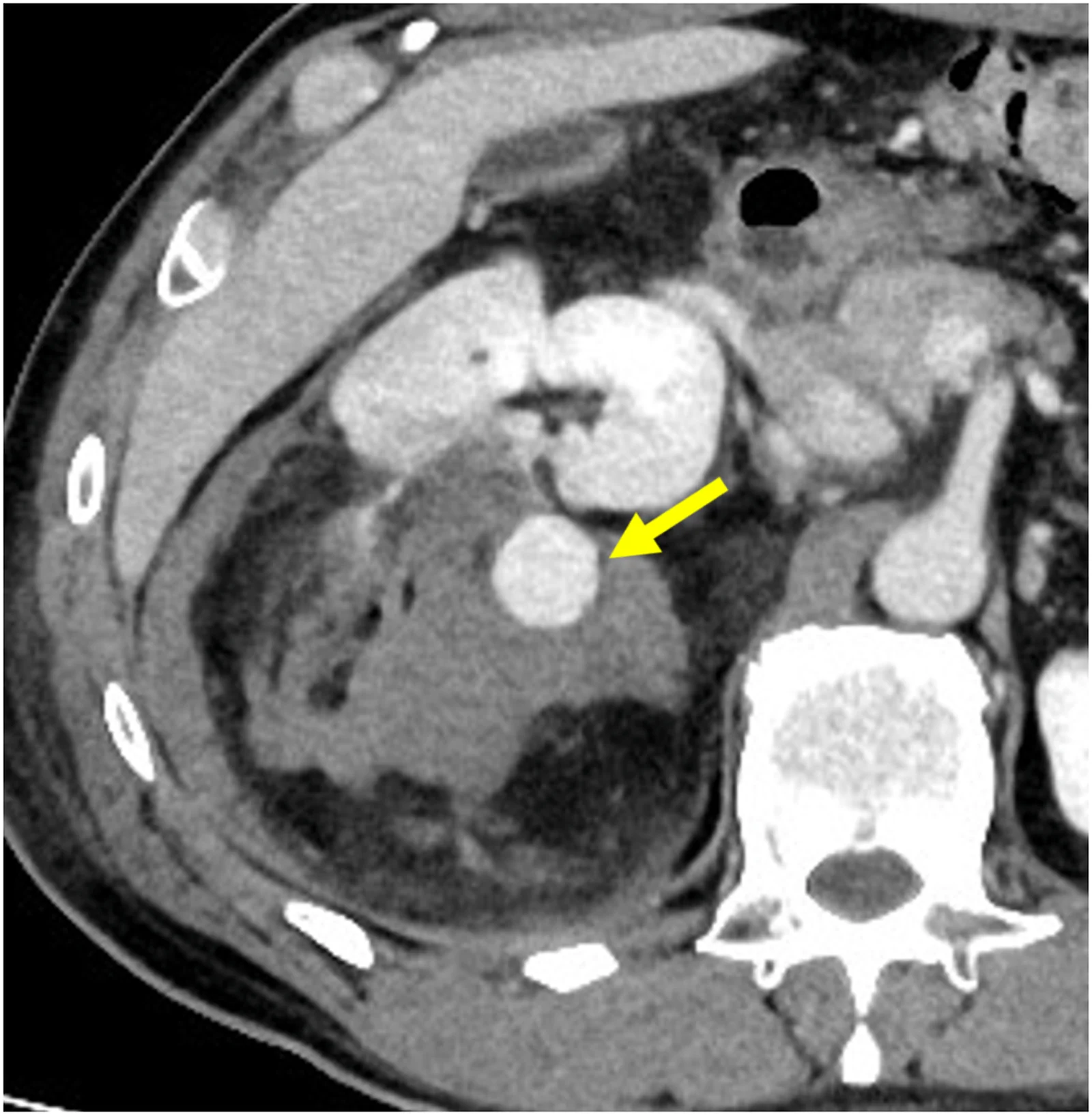
Follow-up contrast-enhanced CT on day 5 after the initial embolization. Axial contrast-enhanced CT image obtained 5 days after the initial embolization demonstrating a newly apparent 20-mm pseudoaneurysm distinct from the lesion identified on the initial CT (arrow).

Emergent transarterial embolization (TAE) was performed for hemorrhage control. Right renal angiography demonstrated tumor staining supplied by the distal segment of a ventral renal arterial branch, with an associated suspected pseudoaneurysm (Fig. 3a). Superselective catheterization of the pseudoaneurysms was achieved using a 2.6-Fr microcatheter (Masters HF; Asahi Intec, Japan) in combination with a 1.9-Fr microcatheter (Carnelian MARVEL; Tokai Medical Products, Japan) in a coaxial configuration. Embolization was performed using gelatin sponge. Post-embolization angiography demonstrated marked reduction in tumor staining within the targeted vascular territory and complete loss of opacification of the suspected pseudoaneurysm (Fig. 3b). No procedure-related complications occurred. The postprocedural course was initially uneventful, with no recurrent symptoms or decline in hemoglobin level, and there was no clinical suspicion of rebleeding. Nevertheless, because the patient had experienced spontaneous rupture without an obvious precipitating factor in the absence of anticoagulant or antiplatelet therapy, together with formation of a large hematoma, follow-up contrast-enhanced CT was performed 5 days after the initial TAE as a precautionary reassessment for residual or additional vascular lesions. The examination demonstrated a newly apparent 20-mm pseudoaneurysm located medial to the pseudoaneurysm identified on the initial CT (Fig. 4). Although the lesion arose within the same ventral branch system, it was considered to originate from a separate subbranch.

**Fig. 5:**
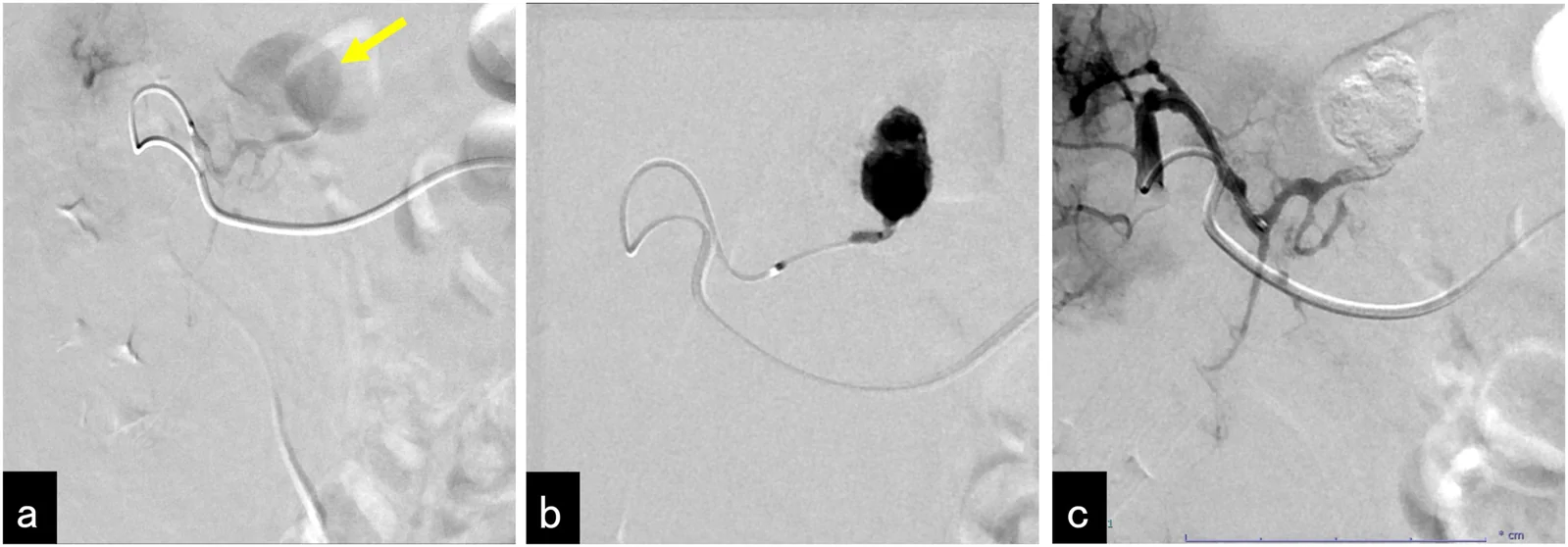
Second angiography and embolization of the newly identified pseudoaneurysm. (a) Selective angiography of a branch distinct from that embolized during the initial procedure demonstrated a pseudoaneurysm (arrow). (b) The pseudoaneurysm was embolized by injection of an NBCA–Lipiodol mixture (NBCA:Lipiodol = 1:4 [20%]) through a microcatheter. (c) Post-embolization angiography confirmed complete obliteration of the pseudoaneurysm.

Given the substantial risk of rupture, repeat TAE was undertaken. During the second procedure, superselective catheterization was performed using a 2.6-Fr microcatheter (Masters HF; Asahi Intec, Japan) and a 1.9-Fr microcatheter (Carry Leon UX19; UTM, Japan) in a coaxial system. Embolization was achieved using an n-butyl cyanoacrylate (NBCA)-Lipiodol mixture (NBCA:Lipiodol = 1:4, 20%) extending from the pseudoaneurysm to the distal feeding artery, and additional microspheres (300–500 μm) were administered to the territory treated during the initial embolization (Fig. 5). Post-embolization main renal angiography confirmed complete obliteration of the pseudoaneurysm. No procedure-related complications were observed.

Follow-up contrast-enhanced CT obtained 2 weeks after repeat TAE demonstrated no new vascular lesions, no evidence of rebleeding, and no deterioration in renal function. Subsequent genetic testing established a diagnosis of tuberous sclerosis complex. The patient was therefore referred for multidisciplinary evaluation and long-term surveillance.

## 3 Discussion

Although AMLs are histologically benign neoplasms, their abnormal vascular architecture confers a clinically significant risk of retroperitoneal hemorrhage.^1^ Because rupture risk increases with lesion size, a tumor diameter of ≥4 cm has historically been used in clinical practice as a pragmatic threshold for intervention.^1^ However, tumor size alone is an imperfect predictor of hemorrhagic risk, and increasing evidence suggests that the presence and diameter of intratumoral aneurysms, including pseudoaneurysms, provide superior risk stratification.^2^ Yamakado et al. demonstrated that an intratumoral aneurysm diameter of ≥5 mm predicts rupture with greater specificity than a tumor diameter of ≥4 cm and identified aneurysm size as the variable most strongly associated with rupture on multivariate analysis.^2^ In the present case, the ruptured 10-cm lesion, concomitant presence of additional large ipsilateral AMLs, and detection of a pseudoaneurysm on initial CT collectively indicated a markedly high-risk hemorrhagic morphology.^1^^,^^2^ Transarterial embolization is a well-established kidney-preserving treatment for ruptured AML and has demonstrated favorable long-term efficacy with preservation of renal function.^3^^,^^5^ In a single-center series of 79 patients, TAE achieved high clinical success, significant tumor size reduction, and minimal long-term deterioration in renal function.^5^ Nevertheless, repeat intervention after embolization remains clinically relevant. In a cohort with 10-year follow-up, the repeat embolization rate reached 41.1%, highlighting that reintervention is a common component of long-term AML management.^4^ Furthermore, retrospective analyses of both symptomatic and asymptomatic AMLs have shown that intratumoral aneurysms are disproportionately represented among lesions associated with hemorrhage and among those requiring subsequent procedures.^6^ The most distinctive feature of the present case is that, despite technically successful superselective TAE with gelatin sponge and an initially stable postprocedural course characterized by stable hemoglobin levels and absence of recurrent symptoms, follow-up CT on day 5 demonstrated a newly apparent 20-mm pseudoaneurysm arising from a separate subbranch within the same ventral arterial system, thereby necessitating repeat embolization. In the acute hemorrhagic setting, procedural priority is appropriately directed toward rapid hemodynamic stabilization; however, comprehensive interrogation and embolization of the entire lesional vascular network may be technically challenging, particularly in large or complex AMLs with multifocal arterial supply. As a result, incomplete treatment of occult feeding vessels may predispose to early rebleeding or delayed identification of residual high-risk vascular lesions.^7^ Indeed, prior reports have documented rebleeding shortly after urgent TAE for ruptured AML requiring repeat embolization, indicating that acute technical success does not necessarily ensure short-term procedural durability.^7^^,^^8^ This case therefore demonstrates that clinically occult vascular lesions warranting reintervention may become radiographically evident despite apparent clinical stability, with important implications for post-embolization surveillance strategies. Assessment of embolization efficacy should likewise extend beyond measurement of overall tumor size. Following embolization, the angiomyogenic component of AML typically regresses, whereas the fatty component may persist; therefore, treatment response should be evaluated with attention to vascular characteristics and enhancing components rather than size reduction alone.^9^ In the present case, the newly identified pseudoaneurysm was treated using an NBCA–Lipiodol mixture (NBCA:Lipiodol = 1:4, 20%), with additional microspheres administered to the previously embolized territory. Because NBCA–Lipiodol embolization has been reported to be both safe and effective in the treatment of AML, this approach may be particularly suitable when durable vascular occlusion is desired and the risk of rerupture is substantial, such as in pseudoaneurysmal lesions.^10^ Importantly, smaller lesions should not be presumed to be clinically innocuous. Even AMLs that do not meet conventional size thresholds for intervention may rupture during surveillance, particularly when vascular-rich morphology is present.^11^ Therefore, hemorrhagic risk assessment should incorporate vascular imaging characteristics in addition to gross morphologic features such as lesion size and multiplicity.^1^^,^^2^

In conclusion, this case demonstrates that in ruptured AMLs with high-risk morphologic characteristics, additional vascular lesions supplied by alternative feeders may become apparent within a short interval even after apparently successful initial embolization and clinical stabilization. This single case does not establish a role for routine early CT after TAE; however, early contrast-enhanced CT may be considered selectively in patients with concerning hemorrhagic or vascular features, even when they remain clinically stable. Further studies are needed to determine whether routine early CT after TAE improves the detection of clinically occult vascular lesions or clinical outcomes.

## CRediT authorship contribution statement

**Takaaki Mori:** Writing – review & editing, Writing – original draft, Visualization, Investigation, Data curation, Conceptualization. **Hajime Takaki:** Supervision. **Yasunari Yamada:** Writing – review & editing, Supervision. **Yoshiyasu Sato:** Resources. **Tadamasa Shibuya:** Resources. **Yoshiki Asayama:** Writing – review & editing, Supervision.

## Ethics declaration

Written informed consent to take part in the study and to publish the article has been obtained from all participants or their legal representatives. The privacy rights of participants have been observed.

This study was performed in compliance with relevant laws, regulatory frameworks and guidelines where the research took place. Ethics committee approval was not required under relevant laws and institutional guidelines. Ethics approval was not required for this single case report in accordance with the policy of our institution.

This research follows the CARE guidelines and the CARE checklist.

## Consent for publication

Written informed consent was obtained from the patient for publication of this case report and accompanying images.

## Funding

This research did not receive any specific grant from funding agencies in the public, commercial, or not-for-profit sectors.

## Declaration of competing interest

The authors declare that they have no known competing financial interests or personal relationships that could have appeared to influence the work reported in this paper.
